# Association between menopausal status and physical function: A systematic review protocol

**DOI:** 10.1371/journal.pone.0280786

**Published:** 2023-01-24

**Authors:** Pedro Macêdo, Ananília Cavalcante, Sabrina Fernandes, Maithê Salustiano, Mateus Lima, Javier Jerez-Roig, Saionara Câmara

**Affiliations:** 1 Postgraduate Program in Physiotherapy, Federal University of Rio Grande do Norte, Natal, Rio Grande do Norte, Brazil; 2 Postgraduate Program in Rehabilitation Sciences, Faculty of Health Sciences of Trairi, Santa Cruz, Rio Grande do Norte, Brazil; 3 Research Group on Methodology, Methods, Models and Outcomes of Health and Social Sciences (M_3_O), Faculty of Health Sciences and Welfare, University of Vic-Central University of Catalonia (UVic-UCC), Barcelona, Spain; CU Anschutz: University of Colorado - Anschutz Medical Campus, UNITED STATES

## Abstract

**Introduction:**

Physical function is considered an important marker of adverse health outcomes. Postmenopausal women seem to have worse physical function, but conflicting results have been reported in the literature. The aim of this systematic review is to assess the association between menopausal status and physical function in community-dwelling women.

**Methods:**

Cross-sectional and/or longitudinal studies which objectively or subjectively assess physical function at different menopausal stages will be included. Studies conducted in institutionalized populations or with any specific medical condition that may have induced menopause (i.e. cancer or degenerative diseases) will be excluded. This systematic review protocol follows the recommendations of the Preferred Reporting Items for Systematic Review and Meta-Analysis Protocols (PRISMA-P). The searches will be carried out in the Pubmed, Embase, SciELO (Scientific Electronic Library Online), LILACS (Latin American and Caribbean Literature on Health Sciences), VHL (Virtual Health Library), Scopus and Web of Science databases, using the search equation “Menopause AND (Physical Performance OR Function)”. The Quality Assessment Tool for Observational Cohort and Cross-Sectional Studies will be considered to assess the methodological quality of the included studies. The selection and evaluation of the methodological quality of the studies will be carried out by independent researchers and the discrepancies will be resolved by a separate researcher.

**Ethics and disclosure:**

Ethical approval is not required as this is a study using secondary data. The results will be published in a scientific journal. We intend to contribute to the expansion of knowledge regarding physical function of women according to the menopause status, thus helping in the perspective of improving health and functioning. This systematic review started in January 2022 and all steps are expected to be finished by October 2022.

**PROSPERO registration number:**

CRD42021289899.

## Introduction

Physical function in human beings is fundamental to health, being marked by the ability to perform daily tasks, including sitting, getting up and walking from one place to another [1]. It can be objectively evaluated by assessing the participant’s ability to perform tasks such as getting up from a chair and walking, or subjectively by assessing the participant’s perception of performing their daily living activities such as climbing stairs or walking around. These tasks are constantly involved in people’s routine and depend on considerable physical performance to perform them. Physical function tends to decline in the aging process and is considered an important predictor of adverse health outcomes throughout life [2]. In the case of women, the functional decline inherent to the aging process seems to start at younger ages when compared to men [3, 4], around age 50, which is a period that coincides with the onset of menopause. It is in this time window that women experience the menopausal transition, determined by the passage from the reproductive phase to the ovarian failure stage [5]. This period is characterized by intense biological, physical and psychological changes resulting from hormonal decline [6]. It is therefore suggested that this hormonal change is responsible for the occurrence of unfavorable outcomes in physical function found in postmenopausal women, including lower grip strength, low gait speed and worse balance [1, 7].

Reproductive life can be classified into three stages and defined by hormonal changes and the regularity of menstrual cycles [8]. For example, pre-menopause corresponds to the normality period of female sex hormone serum levels and monthly presence of the menstrual cycle [8]. Peri-menopause is marked by hormonal and menstrual cycle variation, while post-menopause is defined by the hypoestrogenism stage and absence of menstruation after one year [9].

It is believed that the more accentuated reduction in the estrogen levels of the post-menopause period produces damage to the musculoskeletal system, reducing mass, muscle strength and bone density [10, 11], which can affect women’s physical function and health. However, there are still conflicting results in the literature. While some authors have reported significant differences in physical function measures between women from different menopausal status [1, 12], others fail to present the similar results [13]. Differences in the physical function variables investigated and the socioeconomic context in which the studies are conducted may be factors influencing the divergent results [14]. It has been reported that unfavorable socioeconomic conditions over the life course, such as lower income and low education attainment, can lead to a greater burden of disease [15], and to worse physical performance in older age [16]. Therefore, women who live in contexts with greater economic and social adversities may show greater declines in physiological reserves, making them reach menopause with less intrinsic capacity, and in turn present greater declines in physical function.

A previous literature review conducted by this team [14], which only included studies with postmenopausal women, found that menopause can have long-term effects on physical function, since younger ages at menopause were associated with worse physical function results. Nevertheless, the results were not consistent among the studies included in the review and they varied according to the physical function measure, the context in which the study was developed, and the mean age of the participants included. In this review, we intend to assess if the transition from pre to postmenopausal status is associated with worse physical functioning, which can give us an idea of the short-term effects of menopause on this outcome.

Considering that physical function can significantly impact health during the aging process, understanding if it can be associated with menopausal status is important to identify populations at risk of health adverse outcomes. Also, understanding how menopausal status can influence midlife physical function may provide clues to the origins of frailty and disability among older women, and further help to identify when interventions to prevent disability must be implemented. Furthermore, since the sociocultural reality can interfere with the functional and health condition of individuals [15], understanding if different socioeconomic contexts can impact the associations between menopause and physical function may also help identify in which contexts preventive strategies might be more relevant. Given the conflicting existing literature about the associations between menopausal status and physical function, a systematic review will be useful to understand how these variables associate, and if the context or other methodological aspects may influence these divergences. Therefore, this study aims to analyze the association between menopausal status and physical function through a systematic review. The hypothesis is that post-menopausal women have worse physical function compared to pre- and/or peri-menopause. As a secondary objective, we aim to evaluate the role of socioeconomic context in this association.

## Methods

### Study design

This protocol follows the Preferred Reporting Items for Systematic Review and Meta-Analysis Protocols (PRISMA-P) guide [17]. It was registered in the International Prospective Register of Systematic Reviews (PROSPERO) under the name “Menopausal status and physical function in women: a systematic review” (CRD42021289899).

The key components referring to the study population, investigated exposure, comparator group(s) and main outcome were considered in developing the research question, resulting in the following question: “Is physical function of postmenopausal women worse than pre and/or perimenopausal women, independently of age?”.

### Eligibility criteria

#### Types of studies

Studies published in peer-reviewed scientific journals, which have an observational design, whether cross-sectional or longitudinal, and have classified women at different menopausal stages and compared them in terms of physical function will be included. Experimental studies will not be included since our objective is to understand how menopause, which is a natural process of women’s lives, impact their physical function during middle age.

#### Types of participants

Community-dwelling women and older women. Therefore, studies only including institutionalized women or those that do not specify the results for community-dwelling women will be excluded. Institutionalized women, particularly during middle-age, are likely to present severe adverse health conditions which could confound the associations between menopause and physical function.

#### Exposure

The post-menopause status (natural or surgical) will be considered as exposure, being compared to the pre- and peri-menopause statuses. Thus, studies only including women from pre- and peri-menopause statuses will not be included in this study.

#### Primary outcome

The outcome variable of this study is physical function, whether it is measured through questionnaires about the difficulty of performing functional activities or activities of daily living, or through objective tests such as strength of a specific muscle group, gait speed, chair stand test, static balance, Timed Up and Go test (TUG), Short Physical Performance Battery (SPPB) or the six-minute walk test.

### Strategy for identifying the studies

A search will be carried out in the Pubmed, Embase, ScieELO (Scientific Electronic Library Online), LILACS (Latin American and Caribbean Literature on Health Sciences), Virtual Health Library (BVS), Scopus and Web of Science databases. The search strategy will consider the following standard formula: ‘‘menopause and physical and (performance or function)”. No limits by language or year of publication will be applied. If there is an eligible manuscript in a language other than Portuguese, English or Spanish, it will be sent to a translation service.

### Other search forms

A manual search will be conducted in the reference list of all included articles.

### Data collection and analysis

#### Selection of relevant studies

After removing duplicates, the first step in selecting studies will be performed by reading the titles and abstracts, applying the eligibility criteria by peers independently and blindly by two pairs of researchers (SGGF and ARSC, MDAL and MAS). Articles which meet the inclusion criteria in the first stage will be directed to read the full text and evaluated to confirm eligibility by the same peers independently and blindly by the same researchers in the previous stage. If there is disagreement in this last phase, a separate researcher will resolve them (PRSM). The Rayyan platform (Doha, Qatar) [18] will be used in this process.

#### Data extraction and treatment

Two pairs of researchers (SGGF and ARSC, MDAL and MAS) will independently and blindly extract data from selected articles. If there is a discrepancy in the information collected in the data extraction, a separate researcher (PRSM) will resolve it.

The main data to be extracted will be first/main author, year of publication, country, study design, sample size, menopausal stage groups, sample size by menopausal stage group, type of menopause for postmenopausal women, mean age and standard deviation of sample and groups, inclusion and exclusion criteria, instruments used to assess physical function, values of physical function, differences or measures of association between menopausal groups in relation to measures of physical function and confounding variables.

### Evaluation of the methodological quality of the studies

The methodological quality assessment will be carried out by the same peers as in the previous phases using the Quality Assessment Tool for Observational Cohort and Cross-Sectional Studies [19]. A separate researcher (PRSM) will verify the assessments and resolve discrepancies if any arise.

### Data synthesis

Tables and figures will be used to summarize the results of the studies. A flowchart will illustrate the amount of search results in the literature, and selection of articles at each stage until the final number is found. The collected data will be descriptively analyzed, and a table will provide an overview of the articles included. In addition, the RevMan 5 software program will be used if a metaanalysis is feasible [20]. Heterogeneity will be determined by the I^2^ statistic. A 95% confidence interval and p-values will be considered in these analyses. If sufficient studies are found, subgroup analysis will be performed according to type of menopause (natural or surgical), geographic location and socioeconomic context of the studies. For this, we will extract information about the country where the study was conducted and will group them according to geographic location (e.g., North America, Latin America, Europe, etc.,) and according to their classification of economic development (low-, middle- or high-income country). Descriptive statistics summarizing the results according to the context as well as a subgroup meta-analysis will be performed. The researchers PRSM, SGGF, JJ and SMAC will be involved at this stage.

## Discussion

Although physical function has great relevance for women’s health, few studies have investigated this association, and there are still no results which support major guidelines and recommendations for this population. Understanding the relationship between physical function in the menopause stages is crucial to plan, propose and implement action strategies with a focus on minimizing and delaying functional decline related to the aging process and menopausal transition.

This systematic review will therefore serve as a basis to guide knowledge about physical function at different levels of menopause, because it aims to conduct a comprehensive work that encompasses all the published material, managing to group the functional condition of women who will go through, are experiencing, and those who are already in the post-menopause period. The potential heterogeneity and methodological quality of the articles to be included can limit this systematic review and meta-analysis. Also, the evaluation of the role of socioeconomic context in the association between menopausal status and physical function can be compromised since the country-level socioeconomic classification may not be a sensitive measure of the individual socioeconomic status. This can limit the full understanding of the impact of the socioeconomic status on this association.

## Strengths and limitations of the study

- This systematic review protocol follows the guidelines of the Preferred Reporting Items for Systematic Review and Meta-Analysis Protocols.
- This review will include a comprehensive assessment of the association between physical function and the different menopause stages.
- It can also contribute to understanding how the socioeconomic context can impact this association.
- Heterogeneity of instruments to assess physical function, criteria to identify menopausal stages and confounding variables may limit quantitative analyses, including meta-analysis.
- The country-level socioeconomic classification may not be a sensitive measure of the individual socioeconomic status and limit the understanding of the role of the socioeconomic status on this association.

## Supporting information

S1 Checklist Checklist PRISMA-Preferring to the manuscript. (DOC)
